# Fuller’s Suprapubic Prostatectomy

**Published:** 1905-11

**Authors:** 


### Fuller's Suprapubic Prostatectomy.

IN THE April, 1905, issue of the *Annals of Surgery*, Dr. Eugene Fuller, of New York, published an article entitled "The question of priority in the adoption of the method of total enucleation suprapubically, of the hypertrophied prostate," in which he claimed to be the originator of this particular type of operation and charged Mr. P. J. Freyer of London, with appropriating the operation and giving to it his own name. Mr. Freyer has not seen fit to enter a defense or even to reply to the accusation in six months which have elapsed—whether from a mistaken sense of dignity assailed or that false sense of security which dominates the amateur "Raffles." This being the case it needs must be assumed that he has no defense, and it becomes a duty to place before the medical public the facts in what may in gentle thought be considered a most unwarranted assumption of another man's works.

In his article Fuller shows that he devised the operation and did it in 1894; published it in 1895 in the June issue of the *Journal of Cuntaneous and Genitourinary* diseases in an article entitled "Six successful and successive cases of prostatectomy;" that Freyer was made acquainted with the details of the operation by Dr. Ramon Guiteras and first performed it in 1900, later publishing it as his own operation. Fuller's original article was reprinted in his book on genitourinary diseases published in the spring of 1900. In August of the same year Dr. Guiteras read a paper on "The present status of the treatment of the prostatic hypertrophy in the United States" before the International Medical Congress in Paris and reported his own modification of the Fuller operation, which in brief is that he used finger pressure within the rectum to force the prostate upward while Fuller made pressure against the perineum with his fist. When en route to the Paris meeting Guiteras stopped in London and explained the operation to Freyer, subsequently writing that Freyer had never done such an operation; in fact had not expressed any knowledge that such an operation had been done, and that he would do it at the first opportunity. On November 21, 1900, at St. Peter's hospital Freyer did his first suprapubic prostatectomy, following exactly the method taught him by Guiteras, including Guiteras's modification of the Fuller operation. On June26, 1901, Freyer in a clinical lecture before the Medical Graduates College, of London, calmly assumed credit for the operation as modified by Guiteras and with the latter's friendly visit fresh in his memory refrained from mentioning even his name in connection with the work. Freyer's bold grabbing of everything in sight was little less than disgraceful and several English surgeons took him to task in the columns of the *British Medical Journal.*

To one of them, Mr. Mayo Robson, of Leeds, Freyer replied rather tartly in a spirit of injured pride it seemed, but said nothing regarding a defense of his claims as the originator of the operation. To this Mr. Robson wrote a reply calling attention to the illustrations in Fuller's article of 1894 "which are very much like the drawings in Mr. Freyer's paper." Robson took pains to make his opinion of Freyer understood and left him stripped naked and exposed to the view of the medical world as a bare-faced appropriator of another man's originality. The consequences of the exposure were rather widespread. Keegan, whose litholopaxy work stands foremost in the literature of the subject, in the first flush of Freyer's announcement, warmly congratulated him on his discovery in a letter which was published in the *British Medical Journal.* Later he voluntarily after investigation, stated in the same journal that Fuller had anticipated Freyer by some years and publicly withdrew his congratulations, which was a manly thing to do and more or less of a strain on friendship, when one considers that Freyer had been a member of the Indian medical service in which Keegan was so distinguished a worker. With this mass of evidence arrayed against him and in the absence of definite reply by Mr. Freyer, either to the protests of the English surgeons or to Fuller's caustic article, one must reach the conclusion that in the picturesque language of today the distinguished surgeon at St. Peter's has "been caught with the goods" and that he has no defense.

Of what use then for him to plead guilty even were he so minded. It would not make his sentence the lighter and he could get litle relief by throwing himself on the mercy of the court. There must have been something especially appealing in the Fuller operation to have it snapped up so eagerly by the surgeon of St. Peter's, else he might just as well have taken unto himself Nicholl's way, or Andrews's or McGill's, all of whom devised methods of enucleating the prostate at about the same time that Fuller came to the front with his suprapubic opening. Those who know Dr. Guiteras can well understand why Freyer chose the Fuller operation with the Guiteras modification and labeled it his own. Dr Guiteras is a most interesting talker, he is intensely in earnest; he is descriptive and it is easily seen how luminously simple his modification of Fuller's operation must have looked to Freyer when it was described to him by Guiteras. If Guiteras had taught him one of the other methods this question of Fuller's priority would never have come up and either Nicholl, Andrews or McGill would have been snatching the fruits of their labors from the grip of Freyer.

The JOURNAL has waited full half a year for Freyer to make reply and learns from London that he considers it beneath his dignity to answer the charges which have been brought against him. There never was any question as to where the credit for the operation rightfully belonged; that goes to Fuller; but there was a lingering hope that Freyer would make some explanation which at least would bear some faint semblance of honesty. He has not done so and regrettable as it is he stands convicted of shamelessly attaching his name to an operation to which he had no moral or professional right.

